# Health-Care Services as a Platform for Building Community Resilience among Minority Communities: An Israeli Pilot Study during the COVID-19 Outbreak

**DOI:** 10.3390/ijerph17207523

**Published:** 2020-10-16

**Authors:** Odeya Cohen, Alaa Mahagna, Asmaa Shamia, Ortal Slobodin

**Affiliations:** 1 Nursing Department, Recanati School for Community Health Professions, Faculty of Health Sciences, Ben-Gurion University of the Negev, P.O. Box 653, Beer-Sheva 84105, Israel or alaamah@post.bgu.ac.il (A.M.); asmaas@post.bgu.ac.il (A.S.); 2 Education Department, Ben-Gurion University of the Negev, P.O. Box 653, Beer-Sheva 84105, Israel; ortalslo@bgu.ac.il

**Keywords:** COVID-19, CCRAM, community resilience, emergency, health-care services, minorities

## Abstract

Background: Previous studies of minority groups in times of emergency have tended to focus on risk reduction or on individual resilience, overlooking the community factors that could be bolstered to promote better health and safety outcomes. The current study aimed to examine the role of health-care services in the perceived community resilience of urban and suburban Arab communities in Israel during the COVID-19 outbreak. Method: The study included 196 adults age 17–76 years, who filled out on-line questionnaires in May 2020; 112 participants lived in an urban community and 84 lived in a suburban community. Community resilience was evaluated using the Conjoint Community Resiliency Assessment Measure (CCRAM), a validated five-factor multidimensional instrument. Results: Residents of the suburban community reported higher community resilience than residents of the urban community. This difference was related to increased preparedness levels and strength of place attachment in the suburban community. Residents of suburban communities were also more satisfied and confident in health-care services than those of urban communities. Regression analysis showed that the satisfaction with primary health-care services, and not community type, significantly predicted community resilience. Conclusions: Our results support the pivotal role of primary health care in building community resilience of minority communities in times of emergency and routine.

## 1. Introduction

The COVID-19 pandemic, like other disasters [1,2,3,4], exposes and exacerbates social, economic, and health-care inequalities [5]. Being in a minority group is associated not only with higher risk of hospitalization and deaths due to the Corona virus [5], but also with lower levels of resilience and well-being [6]. Previous studies of minority groups in times of emergency have tended to focus on risk reduction or on individual resilience, overlooking the community factors that could be bolstered to promote better health and safety outcomes [7]. The aim of the current pilot study was to examine the role of health-care services in the perceived community resilience of urban and suburban Arab communities in Israel during the COVID-19 outbreak.

### 1.1. Community Resilience and the Role of Health-Care Services

Community resilience is a multi-dimensional concept, describing the ability of a community to cope with changes or crises [8,9]. Community resilience usually encompasses a set of interlinked core competencies—economic development, information and communication, social capital, and community competence [10]. While individual resilience is a well-established concept within the field of emergency preparedness, the enhancement of community resilience is a relatively new approach [11,12]. According to the community resilience framework, resilience lies at the intersection of routine and emergency periods [13,14]. As such, resilience-building interventions are often conducted during routines and have a double impact—they facilitate the community’s capacity to deal with emergencies and at the same time strengthen public-health services and health-care outcomes in routine [11]. Community resilience is the community’s capacity to use its resources in order to adapt to a sudden disturbance, and eventually to overcome the disturbance, return to routine, and even improve its pre-disturbance functioning [15]. Community resources include social, political, financial, and cultural infrastructures, as well as social capital and shared values [16,17]. Such resources are inherent to the community and are constantly developed and reproduced through interactions between community members and the social institutions [18].

Strong local public health-care systems are the cornerstone of an effective public health response [19,20]. According to the National Health Security Strategy [21], resilient communities are composed of healthy individuals, families, and communities with access to health care and the knowledge and resources to know what to do and care for others in both routine and emergency situations. Previous studies showed that continuity and availability of health-care services during an emergency situation may have more implications than the provision of medical help [14,22]. For example, a study that examined how citizens evaluate hospital responsibility in times of emergency revealed that there is a high public expectation that hospitals will provide significant nonmedical disaster relief, including family reunification and long-term shelter [22]. Furthermore, the confidence that health-care facilities will be available during emergencies was strongly related to factors of community resilience [14], so that when the public is informed and confident that there will be access to health services after a disaster, the community is more resilient. It is noteworthy that the role of health-care services in predicting community resilience did not differ between healthy and unhealthy people, suggesting that the level of trust in health services is largely independent of the actual use of such services [14].

Studies of the role of health-care services in rural vs. urban communities portrayed a complex picture. While rural residents were found to have more limited access to health-care services than urban residents [23], rural communities were less vulnerable to disasters due to their dense social relationship and interactions, which were based on kin and neighbors [15,24]. Building on interview data with 21 survivors of 11 mass emergencies, Druri et al. [24] suggested that shared identity, which in part resulted from the shared experience of the emergency itself, enhances expressions of solidarity and reduces panic behavior. Similarly, Rapaport et al. [15] found that rural communities and smaller townships, which are characterized by intense relationships between their members, demonstrated higher levels of community resilience than did large cities and towns that were characterized by urbanization and social alienation. These findings suggest that high social cohesion may attenuate the adversities related to social and geographical periphery, thus buffering against the consequences of disasters [25,26].

### 1.2. Community Resilience of Minority Groups 

While all populations are affected by disasters, research findings show that racial and ethnic minorities are more likely to be affected by their physical, financial, and psychological effects [4,27,28,29]. This increased vulnerability was attributed to general factors related to class, such as lower socioeconomic status and disparities in health care, as well as to specific factors related to ethnicity, including language and cultural barriers, community isolation, and distrust of health-care systems [30,31,32].

Despite an increasing awareness for the vulnerability of ethnic minority groups to disasters, research on minority resilience is limited [7,26], primarily because most resilience theories were developed in the individualistic Western world [33]. However, research has emphasized that individual models of resilience are not sufficient and at times may even be harmful [34,35].

Mirroring findings of studies of US minority groups [8,36,37,38], studies conducted in Israel to compare the resilience of Arab minority communities and that of the Jewish dominant group showed that Arab children and adults reported lower resilience and well-being in face of trauma, political violence, and mass casualties [39,40,41]. These studies suggested that members of the Arab minority have fewer economic, political, and socio-cultural resources, making them more vulnerable to adversity [39,42]. Although data on the community resilience of Israeli Arabs is very limited [41], a recent study comparing distress and resilience in Jewish and Arab Israeli citizens during the COVID-19 outbreak revealed that Israeli Arabs reported a higher level of distress and lower levels of individual and community resilience than their Jewish counterparts [6]. The authors suggested that the increased levels of anxiety expressed by the Arab participants could indicate their overall permanent concerns and social inequality, rather than health-related anxiety during COVID-19.

### 1.3. The Current Study

The current study aimed to examine the role of health-care services in the perceived community resilience of urban and suburban Arab communities in Israel during the COVID-19 outbreak.

Israel is a multi-cultural country, with clear ethnic disparities in the use of health-care resources [43]. The Arab minority comprises about 20.95% of Israel’s population and includes several different, primarily Arabic-speaking groups; Muslim Arabs, Bedouin Arabs, Druze Arabs, Christian Arabs, and Circassian Arabs. The Israeli Arabs are mostly Sunni (over 1.2 million people), who reside in small towns and villages mainly in the North of Israel. The majority of these identify themselves as Arab or Palestinian by nationality and Israeli by citizenship [44]. The Muslim Arab population is an ethnically homogeneous group that has a high birth rate, an unusually high level of consanguinity, and a low rate of intermarriage compared with other population groups in Israel [45]. While systematic research on ethnic differences in perceived satisfaction of health-care services is still scarce, studies suggest that Arabs suffer from poorer health status compared to Jews and reported higher number of visits to primary care physician [46,47,48]. 

In the current study, we analyzed two case study communities located in Israel. The first is a midsize town, with a population of 55 thousand; the second—a suburban community with a population of close to 12 thousand. Both communities are part of the Triangles area, a group of Israeli Arab towns and villages in central Israel. Israel has a socialized health-care system in which all citizens are free to choose between four health maintenance organizations (HMOs). All HMOs provide equivalent medical services, based on national health regulations. The urban community in our study was served by two different HMOs. The sub-urban community was served by one HMO.

Data was collected in May 2020, during the COVID-19 outbreak. At that time, the number of confirmed cases in the urban community was 80 per 10,000, compared to 23 confirmed cases (per 10,000) in the suburban community [49]. In the adjacent Jewish city, there were 60 confirmed cases (per 10,000) at that time.

During the time of the study, Arab and Bedouin cities and villages reported a surprisingly low number of cases, raising concerns about underdiagnosis. Generally, Arab minority groups in Israel experienced serious delays in complying with the formal health instructions, resulting in high infection rates and lockdowns of big Arab cities. For example, at the end of March, the ratio of people under quarantine in two large Arab cities was less than 1:1000. A month later, these cities were under local lockdown following a sharp rise in the number of COVID-19 cases [50]. This high infection rate in Arab communities reflects multiple socio-cultural factors including lower socioeconomic status, kinship-based family fabric, high-density housing, distrust in governmental authorities, and limited access to information about the virus [51,52].

Our research hypotheses were:

1. Higher levels of community resilience will be scored in the suburban community, a smaller local council that may be more socially cohesive than the urban community.
2. Community resilience would be positively associated with increased confidence and satisfaction with health-care services (primary and specialized).
3. Community type would play a unique role in community resilience, over and above the perceptions of health-care services as a provider of medical treatment.

## 2. Materials and Methods 

### 2.1. Study Design, Setting, and Participants

The study was a cross-sectional study conducted among residents of two Arab communities in Israel one urban, the other sub.

Data was collected in May 2020. Eligibility criteria for study inclusion were: (a) Age above 17 years, (b) ability to understand and complete self-report questionnaires, and (c) living and receiving health-care services in the investigated communities. Electronic questionnaires using a web-based survey software (Qualtrics; www.qualtrics.com), were sent out through social networks. To have adequate power to detect a medium effect size in multiple regression, using a two-tailed test, with three predictors, α = 0.05, and power = 0.80 [53], a minimum of 76 participants was required.

### 2.2. Instruments

Background variables included age, gender, education level, marital status, religious affiliation, number of children, and employment. Community resilience was assessed by the short version of the Conjoint Community Resiliency Assessment Measure (CCRAM-10; [54]), which is a self-report questionnaire with ten items covering five significant constructs of community resilience: Leadership, Collective Efficacy, Preparedness, Place Attachment and Social Trust. The questionnaire also addresses demographic details and information on relevant personal experience of the respondent. Responses are rated on a 5-point Likert scale. The CCRAM-10 was found to be reliably capable of assessing the original five factors [55]. 

Self-rated health (SRH) is a simple, easy to administer measure of general health [56], which was initially developed to replace clinical assessments in survey research [57]. SRH is typically measured as a single item, the most common wording of which is “In general, would you say your health is” with the response items “excellent,” “very good,” “good,” “fair,” or “poor”. 

Satisfaction and confidence in primary, secondary, and national health-care services: The questionnaire was based on a survey instrument developed and used by the Myers-JDC Brookdale Institute (MJBI) for a national satisfaction survey typically conducted every 2–3 years [58]. It consists of about 70 questions with sections that focused on access to health care, patient satisfaction, health services use, branch and staff hours, health status, and demographics. Respondents were asked to rate their level of satisfaction for a variety of services as “very satisfied,” “satisfied,” “not so satisfied,” or “not satisfied”.

### 2.3. Data Analysis

First, descriptive statistics were analyzed for all study variables, by community type. Next, the variables for the two communities were compared, using a multivariate analysis of covariance (MANCOVA). Demographic variables that differed between the two study groups, including age, education level, marital status, and level of religiosity, served as covariates. Furthermore, correlational analysis examined the relationships between community resilience and satisfaction and confidence with health-care services. Finally, the roles of community type, satisfaction and confidence with health-care services in predicating community resilience were examined using hierarchical linear regression analyses. In the regression analyses, participants’ age, gender, and health status were entered at Step 1, community type at Step 2, and the three health-care measures (satisfaction with primary services, satisfaction with specialized services and confidence) at the last step. Data analyses were carried out using IBM SPSS (the statistical package for the social sciences), version 26 [59].

### 2.4. Ethical Approval

Ethical approval was obtained from the Institutional Review Board (IRB) of the Faculty of Health Sciences at Ben-Gurion University of the Negev (13-2020). Respondents were informed that participation was voluntary without remuneration and that the responses would be collected and analyzed anonymously.

## 3. Results

### 3.1. Demographic Characteristics of Study Participants

The study included 196 adults (63% females), of whom 112 lived in the urban community and 84 lived the suburban one. Participants ranged in age from 17 to 76 (mean age = 35.03 years, *SD* = 14.34). Most of the participants were married (60%). Table 1 presents a comparison between the two study communities in background and study variables. *T* test for independent samples and Chi-Square tests revealed that the suburban community was older and more religious than the urban one. No differences between the two communities were observed in the distribution of gender and education level.

### 3.2. Community Resilience and Community Type

After controlling for age, education level, marital status, and level of religiosity, a MANCOVA showed that, in line with our first hypothesis, the community type had a significant effect on overall community resilience: *F* (5,168) = 3.145, *p* = 0.010, Pillai’s Trace = 0.86.

**Figure 1:**
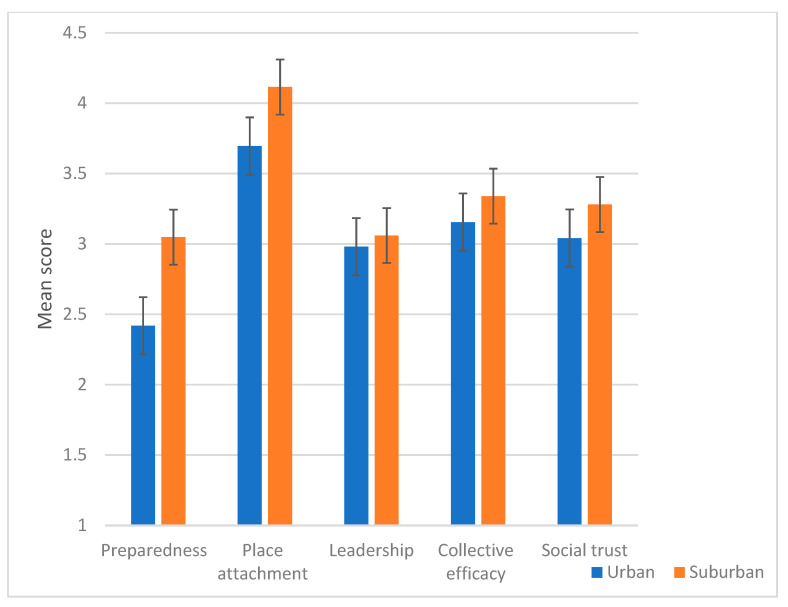
Differences between members of urban and suburban communities in the CCRAM factors.

As seen in Figure 1, members of the suburban community were significantly higher on their preparedness level *F* (1,178) = 11.604, *p* = 0.001 and the strength of place attachment *F* (1,178) = 4.332, *p* = 0.39 than members of the urban community.

No differences between community types were identified in leadership, *F* (1,178) = 0.645, *p* = 0.423; collective efficacy *F* (1,178) = 0.950, *p* = 331; and social trust *F* (1,178) = 1.130, *p* = 0.289.

### 3.3. The Role of the Health-Care Services in Predicting Community Resilience 

As a first step, a correlation matrix was conducted. Table 2 presents Pearson correlations between study variables. Supporting the second hypothesis, results showed that community resilience was positively associated with satisfaction with primary health-care services (*r* = 33, *p* < 01), satisfaction with specialized health-care services (*r* = 28, *p* < 0.1), and confidence in health-care services (*r* = 18, *p* < 0.5), but not with self-rated health (*r* = 0.8, *p* > 0.5).

Results of the regression analysis, presented in Table 3, showed that satisfaction with primary health-care services was the only predictor of community resilience (β = 0.31, *p* < 0.1). 

## 4. Discussion

The aim of the current study was to examine the role of health-care services in the perceived community resilience of urban and suburban Arab communities in Israel during the COVID-19 outbreak. Our results showed that residents of the suburban community reported higher community resilience than residents of the urban community, and that this difference was related to increased preparedness levels and strength of place attachment in the suburban community.

Members of suburban communities were also more satisfied and confident in health-care services than those of urban communities. However, when examining the relative contribution of these variables to the variance in community resilience, only satisfaction with primary health-care services, and not community type, had a significant contribution.

Our results are in line with previous studies in non-minority groups [15,55], which demonstrated an inverse relationship between the perceived community resilience and community size. For example, in two studies involving small to medium size Jewish towns in Israel during non-emergency periods, members of small urban communities and local authorities reported lower community resilience compared to villages, planned communities, and collective communities [54,55]. Recently, Rapaport et al. [15] found that rural communities showed the highest levels of community resilience factors, followed by suburban and then urban communities. These findings suggested that rural communities translate their strong social resources, such as shared ideology, close relationship, and social homogeneity, into perceived resilience.

While these studies focused on the role of socio-cultural resources in explaining community resilience, the current study focused on the role of perceived health-care services. Specifically, our results showed that members of the suburban community reported higher satisfaction and confidence in health-care services and that a such sense of satisfaction may be one of the underlying mechanisms linking community type and community resilience. These findings are surprising, given that small communities have inferior access to resources and emergency aid [60,61]. One possible explanation for our findings is that perceived high-quality primary health-care services provides participants with a strong sense of security, preparedness and belonging, which may compensate for other disadvantages associated with living in a small town. There is evidence to suggest that primary health-care services play a key role in social, political, and international community development, particularly in low-income minority groups [62,63]. Several studies in minority populations, including Arabs in Israel, have shown that minority groups tend to use primary health-care services more often than non-minority ones, partly due to health literacy [62]. For example, a recent Israeli study indicated that Arabs have reported receiving health promotion advice (e.g., physical activity, diet) from their primary doctor more commonly than Jewish patients [64].

Another possible explanation for the higher satisfaction with health-care services in the suburban community may be related to the type of the current emergency. Previous studies of community resilience were conducted during non-emergency periods [11,15,54] or during mass traumas (e.g., war, terror) [65,66,67], whereas the current study examined community resilience during a health crisis. While this crisis may potentially increase communities’ needs of health services, one consequence of the COVID-19 pandemic has been under-use of important medical services for patients with urgent and emergent health needs that were unrelated to COVID-19 [68,69]. Such decreased load on health-care services may improve the perceived availability of primary and specialized health-care services and contribute to patient–doctor communication, leading to increased satisfaction with health services.

Finally, the key role of primary health-care services in community resilience during the COVID-19 crisis might be explained by the dynamics of community resilience. Studies exploring the changes in perceived community resilience between routine and emergency periods, revealed that community resilience levels were higher during times of emergency and lower in times of non-emergency [66,70]. This increase in community resilience shortly after a disaster is typical of many types of emergencies and reflects a sudden increase in community cohesion. For instance, a study of social cohesion and perceived community resilience in four rural communities in Canada that had experienced wildfires and floods, showed that the link between social cohesion and community resilience became weaker over time [71]. Furthermore, perceived community resilience fluctuated with time following a disaster, showing high scores in the first year, and then a decrease on the second year, followed by increase at the 6th year, and subsequent decrease at the 10th year. Another study which examined fluctuations in community resilience in times of emergency and routine found that at least three factors of community resilience—preparedness, leadership, and collective efficacy—consistently increased in response to an emergency [66]. This dynamics of community resilience indicates that acute disruptions in life are accompanied by a quick and efficient allocation of individual and community resources, such as adequate function of local authorities and emergency services, increased availability of shelters and the public’s awareness, mutual support and solidarity [72]. It is, therefore, possible that due to enhanced social cohesion, suburban communities were more effective in translating their community resources to increased resilience shortly after the COVID-19 outbreak. Nevertheless, the long-term effects of the relative inferiority of health-care services on the community resilience of small communities is yet to be resolved.

Several limitations should be noted when considering the finding in the current study. First, the cross-sectional nature of this study does not allow us to draw any causal relationships between variables. Thus, inferences about the direction of the relationships were based on conceptual rather than empirical considerations. Additionally, our cross-sectional design does not allow the monitoring of changes in community resilience over time. As previous research has shown [66], community resilience tends to increase shortly after disasters and then gradually decrease. Future research should consider the dynamics of community resilience in different types of communities.

A related limitation is that the data collection was restricted to the first wave of the COVID-19 outbreak, shortly after a general lockdown, and a time when the number of COVID-19 cases was limited in both communities, partially due to underdiagnosis. It is, therefore, difficult to ascertain the degree to which the primary health-care services effectively faced the challenges of the first wave how well they addressed the needs of each community. According to the World Health Organization [73], the main principles of primary care in the COVID-19 response should include early identification and management of potential cases, avert the risk of transmission of infection to contacts and health-care workers, maintain delivery of essential health services, enhance existing surveillance (e.g., influenza-like illness), and strengthen risk communication and community engagement. It is crucial for future studies to investigate the capacity of primary health-care services to address these principles and their association with community resilience.

Another limitation of the current study is the exclusive reliance on self-reported data. Incorporating data from local authorities and health services about availability and preparedness would be an effective way of overcoming this limitation. Finally, the sampling method, which was based on snowball sampling and social media networks, may contribute to an unbalanced sample in terms of demographic characteristics and disclosure of personal information [74].

Given the scarcity of research on community resilience of ethnic minority groups, our study provides initial insight into the public-health resources that contribute to community resilience in times of crisis. Our results are consistent with those of previous research [14], and suggest that the central role that primary health-care services play in strengthening communities is far beyond the provision of medical treatment and is independent of individuals’ health status. Although the research on primary health-care services in low-income and middle-income populations is currently scarce and fragmented [75], previous studies have offered several strategies to strengthen primary health-care services in these contexts [76]. Such strategies include the facilitation of intersectoral collaboration between different stakeholders of health-care [77,78], educational interventions for both providers and consumers [79,80], intensive professional training, and health policy support (e.g., equitable payment of primary health care compared with other areas of specialization) [81]. Additionally, incorporating mobile and communication technologies into the services may provide many opportunities to involve patients in managing and monitoring their health as well as to promote collaboration with specialists and facilitating connections with nursing homes and home health [82]. Finally, there is a need for an expanded and more coordinated effort for research focused on primary health-care interventions in order to bridge the evidence policy and practice gaps [83].

Future studies investigating community resilience in cultural and ethnic minority groups should consider the role of culture and context in the development of community resilience. A cultural interpretation of community resilience suggests that the community’s capacity to deal with changes is highly influenced by cultural attitudes, values, and norms. From this point of view, a culturally sensitive approach to emergencies entails a global and local understanding of culture-related expectations and attitudes [84,85]. For example, research in minority communities in Israel during the first wave of COVID-19 showed that keeping social distancing in large, multi-generational households was almost impossible not only due to inadequate space for quarantining but also because shared values make it culturally unacceptable to avoid physical proximity or to send an ill family member away [51].

Although the results of the current study reflect the socio-political context of Israeli Arabs, the vulnerability of ethnic/cultural minority groups to the effects of the COVID-19 outbreak is a public emergency of global concern [5]. Like other deadly pandemics in human history [86,87,88], the COVID-19 intensifies tensions between groups, increasing racism, discrimination, and mass violence against minorities as people seek a scapegoat. Our study thus highlights the role of health-care services as a delivery platform for enhancing the community resilience of minorities and for mitigating existing gaps and inequalities.

## 5. Conclusions

Although the importance of community resilience to public health is well established [89,90], there is no clear consensus about the mechanisms for enhancing community resilience. Focusing on the community resilience in communities of the Arab minority in Israel, our pilot study suggests that high-quality primary health-care services may be a key source of community resilience over and above the role of community type. These results provide further support for the importance of primary health-care services as an accessible resource, particularly for underserved populations. Therefore, interventions tailored to strengthen primary health-care services may serve as a platform for enhancing the social fabric and mitigating social inequity in emergencies as well as in routine.

## Figures and Tables

**Table 1:** Differences in urban and suburban communities in study variables.

Variable		Urban Community (*n* = 112)	Suburban Community (*n* = 84)	Difference
Age *M* (*SD*)		31.46 (15.13)	39.73 (11.89)	*t* (194) = −4.176, *p* = 0.000
Gender	Male	34	27	χ^2^ (1) =0.03, *p* = 0.85
	Female	76	57	
Religious affiliation	Religiously observant.	36	41	χ^2^ (1) =7.04, *p* = 0.03
	Traditional	70	42	
	Secular	6	1	
Years of education	<8	16	7	χ^2^ (1) = 3.40, *p* = 183
	Highschool	29	17	
	Academic	64	59	
Community resilience *M* (*SD*)		3.08 (0.71)	3.37 (0.58)	*F* (1,185) = 3.98, *p* = 0.048
Satisfaction with community health services *M* (*SD*)		3.99 (0.62)	4.12 (0.57)	*F* (1,185) = 7.20, *p* = 0.008
Satisfaction with specialized health services *M* (*SD*)		4.03 (0.73)	4.28 (0.66)	*F* (1,184) = 7.77, *p* = 0.006
Confidence in health services		3.45 (0.69)	3.47 (0.77)	*F* (1,184) = 3.75, *p* = 0.054

**Table 2:** Pearson correlations between main research variables (*N* = 196).

	Age	Gender	Community Type	Satisfaction with Primary Health Services	Satisfaction with Specialized Health Services	Confidence in Health Services	Community Resilience	Self-Rated Health
Age	1	−0.32 **	0.27 **	0.16 *	0.09	0.11	0.09	−0.36 **
Gender		1	−0.01	0.16 *	0.17 *	0.08	0.01	0.11
Community type			1	0.12	0.17 *	0.09	0.19 **	−0.02
Satisfaction with primary healthcare services				1	0.60 **	0.56 **	0.33 **	0.18 *
Satisfaction with specialized healthcare services					1	0.40 **	0.28 **	0.14
Confidence in healthcare services						1	0.18 *	−0.001
Community resilience							1	0.08
Self-rated health								1

* *p* < 0.05; ** *p* < 0.01.

**Table 3:** Multiple regression analyses predicting community resilience by satisfaction and confidence in health-care services.

Step		Unstandardized Coefficients		Standardized Coefficients	*F*	
		*B*	Std. Error	Beta		Adj. *R* Square
1	Age	0.01	0.01	0.12	1.20	0.004
	Gender	0.01	0.14	0.01		
	Self-rated health	0.06	0.04	0.155		
2	Age	0.004	0.01	0.08	1.85	0.20
	Gender	−0.00	0.14	−0.01		
	Self-rated health	0.06	0.04	0.12		
	Community type	0.25	0.13	0.16		
3	Age	0.01	0.01	0.13	4.40 ***	0.124
	Gender	−0.07	0.14	−0.04		
	Self-rated health	0.03	0.04	0.07		
	Community type	0.15	0.13	0.09		
	Satisfaction with primary healthcare services	0.41	0.14	0.31 **		
	Satisfaction with specialized healthcare services	0.08	0.11	0.07		
	Confidence in healthcare services	−0.003	0.10	−0.003		

** *p* < 0.01; *** *p* < 0.001.

## References

[1] Adams V. (2013). Markets of Sorrow, Labors of Faith: New Orleans in the Wake of Katrina.

[2] Elliott J.R., Bellone-Hite A., Devine J. (2009). Unequal return: The uneven resettlements of New Orleans’s uptown neighborhoods. Org. Environ..

[3] Weber L., Messias D.K.H. (2012). Mississippi front-line recovery work after Hurricane Katrina: An analysis of the intersections of gender, race, and class in advocacy, power relations, and health. Soc. Sci. Med..

[4] Davidson T.M., Price M., McCauley J.L., Ruggiero K.J. (2013). Disaster impact across cultural groups: Comparison of Whites, African Americans, and Latinos. Am. J. Community Psychol..

[5] Centers for Disease Control and Prevention COVID-19, 2020. https://www.cdc.gov/coronavirus/2019-ncov/index.html.

[6] Kimhi S., Eshel Y., Marciano H., Adini B. (2020). Distress and Resilience in the Days of COVID-19: Comparing Two Ethnicities. Int. J. Environ. Res. Public Health.

[7] Davis R., Cook D., Cohen L. (2005). A community resilience approach to reducing ethnic and racial disparities in health. Am. J. Public Health.

[8] Patel S.S., Rogers M.B., Amlôt R., Rubin G.J. (2017). What do we mean by ‘Community Resilience’? A systematic literature review of how it is defined in the literature. PLoS Curr..

[9] Magis K. (2010). Community resilience: An indicator of social sustainability. Soc. Nat. Res..

[10] Norris F.H., Stevens S.P., Pfefferbaum B., Wyche K.F., Pfefferbaum R.L. (2008). Community resilience as a metaphor, theory, set of capacities, and strategy for disaster readiness. Am. J. Community Psychol..

[11] Cohen O., Goldberg A., Lahad M., Ahronsonn-Daniel L. (2017). Building resilience: The relationship between information provided by municipal authorities during emergency situations and community resilience. Technol. Forecast. Soc. Chang..

[12] Madsen W., O’Mullan C. (2016). Perception of community resilience after natural disaster in a rural Australian town. J. Community Psychol..

[13] Chandra A., Acosta J., Howard S., Uscher-Pines L., Williams M., Yeung D., Garnett J., Meredith L.S. (2011). Building community resilience to disasters: A way forward to enhance national health security. Rand. Health Q..

[14] Cohen O., Shapira S., Aharonson-Daniel L., Shamian J. (2019). Confidence in health-services availability during disasters and emergency situations-does it matter?-Lessons learned from an Israeli population survey. Int. J. Environ. Res. Public Health.

[15] Rapaport C., Hornik-Lurie T., Cohen O., Lahad M., Leykin D., Aharonson-Daniel L. (2018). The relationship between community type and community resilience. Int. J. Disaster Risk Reduct..

[16] Pickett S.T.A., Cadenasso J.M., Grove M. (2004). Resilient cities: Meaning, models, and metaphor for integrating the ecological, socio-economic, and planning realms. Landsc. Urban Plan..

[17] Pierce J.C.M., Budd W.W., Lovrich N.P. (2011). Resilience and sustainability in US urban areas. Environ. Polit..

[18] Flora C.B., Flora J.L. (2004). Rural Communities: Legacy and Change.

[19] Centers for Disease Control and Prevention PUBLIC Health Preparedness Capabilities: National Standards for State and Local Planning, 2011. https://www.cdc.gov/cpr/readiness/00_docs/DSLR_capabilities_July.pdf.

[20] Redwood-Campbell L., Abrahams J. (2011). Primary health care and disasters-the current state of the literature: What we know, gaps and next steps. Prehosp. Disaster. Med..

[21] Framework for the NHSS Assistant Secretary for Preparedness and Response. https://www.phe.gov/about/aspr/Pages/default.aspx.

[22] Charney R.L., Rebmann T., Esguerra C.R., Lai C.W., Dalawari P. (2013). Public perceptions of hospital responsibilities to those presenting without medical injury or illness during a disaster. J. Emerg. Med..

[23] Wells M. (2009). Resilience in rural community-dwelling older adults. J. Rural. Health.

[24] Drury J., Cocking C., Reicher S. (2009). Everyone for themselves? A comparative study of crowd solidarity among emergency survivors. Br. J. Soc. Psychol..

[25] Weine S., Kulauzovic Y., Klebic A., Besic S., Mujagic A., Muzurovic J., Rolland J. (2008). Evaluating a multiple-family group access intervention for refugees with PTSD. J. Marital. Fam. Ther..

[26] Slobodin O., de Jong J.T. (2015). Mental health interventions for traumatized asylum seekers and refugees: What do we know about their efficacy?. Int. J. Soc. Psychiatr..

[27] Eisenman D.P., Wold C., Fielding J., Long A., Setodji C., Hickey S., Gelberg L. (2006). Differences in individual-level terrorism preparedness in Los Angeles County. Am. J. Prev. Med..

[28] Eisenman D.P., Zhou Q., Ong M., Asch S., Glik D., Long A. (2009). Variations in disaster preparedness by mental health, perceived general health, and disability status. Disaster. Med. Public Health Prep..

[29] Hawkins R.L., Maurer K. (2011). ‘You fix my community, you have fixed my life’: The disruption and rebuilding of ontological security in New Orleans. Disasters.

[30] Bethel J.W., Burke S.C., Britt A.F. (2013). Disparity in disaster preparedness between racial/ethnic groups. Disaster Health.

[31] Guha-Sapir D. (2011). EMDAT and Trends in Natural Disasters.

[32] Pole N., Gone G.P., Kulkarni M. (2008). Posttraumatic stress disorder among ethnoracial minorities in the United States. Clin. Psychol..

[33] Ungar M. (2011). The social ecology of resilience. Addressing contextual and cultural ambiguity of a nascent construct. Am. J. Orthopsychiatr..

[34] Bracken P.J. (2002). Trauma: Culture, Meaning and Philosophy.

[35] Westoby P. (2008). Developing a community-development approach through engaging resettling Southern Sudanese refugees within Australia. Community. Dev. J..

[36] Galea S., Ahern J., Resnick H., Kilpatrick D., Bucuvalas M., Gold J., Vlahov D. (2002). Psychological sequelae of the September 11 terrorist attacks in New York City. N. Engl. J. Med..

[37] Heck N.C. (2015). The potential to promote resilience: Piloting a minority stress-informed, GSA-based, mental health promotion program for LGBTQ youth. Psychol. Sex Orientat. Gend. Divers..

[38] Norris F.H., Sherrieb K., Pfefferbaum B., Southwick S.M., Litz B.T., Charney D., Friedman M.J. (2011). Community resilience: Concepts, assessment, and implications for intervention. Resilience and Mental Health: Challenges across the Lifespan.

[39] Hobfoll S.E., Palmieri P.A., Johnson R.J., Canetti-Nisim D., Hall B.J., Galea S. (2009). Trajectories of resilience, resistance, and distress during ongoing terrorism: The case of Jews and Arabs in Israel. J. Consult. Clin. Psychol..

[40] Kimchi S., Dror G., Sapir S. (2017). Resilience among students from the majority and minority group: The Israeli case. J. Psychol. Behav. Sci..

[41] Marciano H., Eshel Y., Kimhi S. (2019). Predictors of Individual, Community and National Resiliencies of Israeli Jews and Arabs. Int. J. Psychol..

[42] Braun-Lewensohn O., Sagy S. (2014). Community resilience and sense of coherence as protective factors in explaining stress reactions: Comparing cities and rural communities during missiles attacks. Community Ment. Health J..

[43] Nakash O., Nagar M., Danilovich E., Bentov-Gofrit D., Lurie I., Steiner E., Sadeh-Sharvit S., Szor H., Levav I. (2013). Ethnic disparities in mental health treatment gap in a community-based survey and in access to care in psychiatric clinics. Int. J. Soc. Psychiatr..

[44] Middle East Report. IDENTITY Crisis: Israel and Its Arab Citizens, 2004. https://www.crisisgroup.org/middle-east-north-africa/eastern-mediterranean/israelpalestine/identity-crisis-israel-and-its-arab-citizens.

[45] Vardi-Saliternik R., Friedlander Y., Cohen T. (2002). Consanguinity in a population sample of Israeli Muslim Arabs, Christian Arabs and Druze. Ann. Hum. Biol..

[46] Baron-Epel O., Garty N., Green M.S. (2007). Inequalities in use of health services among Jews and Arabs in Israel. Health Ser. Res..

[47] Khatib M. Health of Arab Women in Israel, the Galilee Society—The Arab National Society for Health Research and Services, 2012. https://arab.org/directory/the-galilee-society-the-arab-national-society-for-health-research-and-services/.

[48] Na’amnih W., Muhsen K., Tarabeia J., Saabneh A., Green M.S. (2010). Trends in the gap in life expectancy between Arabs and Jews in Israel between 1975 and 2004. Int. J. Epidemiol..

[49] Ministry of Health (2020). The Novel Coronavirus. https://www.health.gov.il/English/Topics/Diseases/corona/Pages/default.aspx.

[50] Staff T. Checkpoints Dismantled as Lockdown of Virus-Hit Arab Towns in North Lifted. The Times of Israel. 25 April 2020. https://www.timesofisrael.com/checkpoints-dismantled-as-lockdown-of-virus-hit-arab-towns-in-north-lifted/.

[51] Slobodin O., Cohen O. (2020). A culturally-competent approach to emergency management: What lessons can we learn from the COVID-19?. Psychol. Trauma..

[52] Ebenstein R. Fighting Back Against COVID-19 in Israel’s Bedouin Community. Fathom. (1 April 2020). https://fathomjournal.org/fighting-back-against-covid-19-in-the-bedouin-community/.

[53] Cohen J. (1992). Statistical Power Analysis for the Behavioral Sciences.

[54] Cohen O., Leykin D., Lahad M., Goldberg A., Aharonson-Daniel L. (2013). The conjoint community resiliency assessment measure as a baseline for profiling and predicting community resilience for emergencies. Technol. Forecast. Soc. Chang..

[55] Leykin D., Lahad M., Cohen O., Goldberg A., Aharonson-Daniel L. (2013). Conjoint community Resiliency assessment measure-28/10 items (CCRAM28 and CCRAM10): A self-report tool for assessing community resilience. Am. J. Community Psychol..

[56] Bombak A.E. (2013). Self-rated health and public health: A critical perspective. Front. Public Health.

[57] Strawbridge W.J., Wallhagen M.I. (1999). Self-rated health and mortality over three decades: Results from a time-dependent covariate analysis. Res. Aging..

[58] Brammli-Greenberg S., Medina-Artom T., Belinsky A. (2017). Summary of Findings from the Eleventh Survey of Public Opinion on the Level of Service and Performance of the Healthcare System.

[59] IBM Corp (2019). IBM SPSS Statistics for Windows, Version 26.0.

[60] Beggs J.J., Haines V.A., Hurlbert J.S. (1996). Revisiting the rural-urban contrast: Personal networks in nonmetropolitan and metropolitan settings. Rural. Soc..

[61] Lev-Wiesel R. (2003). Indicators constituting the construct of ‘perceived community cohesion’. Community Dev. J..

[62] Van der Gaag M., van der Heide I., Spreeuwenberg P.M.M., Brabers A.E.M., Rasemakers J.D.J.M. (2017). Health literacy and primary health care use of ethnic minorities in the Netherlands. BMC Health Serv. Res..

[63] Uiters E., Deville W., Foets M., Spreeuwenberg P., Groenewegen P.P. (2009). Differences between immigrant and non-immigrant groups in the use of primary medical care; a systematic review. BMC Health. Serv. Res..

[64] Hayek S., Derhy S., Smith M.L., Towne S.D., Zelber-Sagi S. (2020). Patient satisfaction with primary care physician performance in a multicultural population. Isr. J. Health Policy Res..

[65] Kimhi S., Shamai M. (2004). Community resilience and the impact of stress: Adult response to Israel’s withdrawal from Lebanon. J. Community Psychol..

[66] Leykin D., Lahad M., Cohen R., Goldberg A., Aharonson-Daniel L. (2016). The dynamics of Community Resilience between routine and emergency situations. Int. J. Disaster Risk Reduct..

[67] Shamai M., Kimhi S., Enosh G. (2007). Social systems and personal reactions to threats of war. J. Soc. Per. Relatsh..

[68] De Filippo O., D’Ascenzo F., Angelini F., Bocchino P.P., Conrotto F., Saglietto A., Secco G.G., Campo G., Gallone G., Verardi R. (2020). Reduced rate of hospital admissions for ACS during Covid-19 outbreak in Northern Italy. N. Eng. J. Med..

[69] Guo H., Zhou Y., Liu X., Tan J. (2020). The impact of the COVID-19 epidemic on the utilization of emergency dental services. J. Dent. Sci..

[70] Raphael B. (1986). When Disaster Strikes: How Individuals and Communities Cope with Catastrophe.

[71] Kapucu N., Hawkins C.V., Rivera F.I. (2013). Disaster preparedness and resilience for rural communities. Risk. Hazards Crisis Public Policy.

[72] Richards L., Howden S.M. (2012). Transformational adaptation: Agriculture and climate change. Crop. Pasture Sci..

[73] World Health Organization (2020). Role of Primary Care in the COVID-19 Response. https://apps.who.int/iris/bitstream/handle/10665/331921/Primary-care-COVID-19-eng.pdf?sequence=1&isAllowed=y.

[74] Hall M., Mazarakis A., Chorley M., Caton S. (2018). Editorial of the special issue on following user pathways: Key contributions and future directions in cross-platform social media research. Int. J. Hum-Comput. Int..

[75] World Health Organization (2017). World Report on Health Policy and Systems Research. https://www.who.int/alliance-hpsr/resources/publications/worldreport-hpsr/en/.

[76] Haque M., Islam T., Rahman N.A.A., McKimm J., Abdullah A., Dhingra S. (2020). Strengthening primary health-care services to help prevent and control long-term (chronic) non-communicable diseases in low- and middle-income countries. Risk Manag. Healthc. Policy.

[77] Bennett S., Glandon D., Rasanathan K. (2018). Governing multisectoral action for health in low-income and middle-income countries: Unpacking the problem and rising to the challenge. BMJ. Glob. Health.

[78] Rasanathan K., Atkins V., Mwansambo C., Soucat A., Bennett S. (2018). Governing multisectoral action for health in low-income and middle-income countries: An agenda for the way forward. BMJ Glob. Health.

[79] Ramli A.S., Lakshmanan S., Haniff J., Selvarajah S., Tong S.F., Bujang M.-A., Abdul-Razak S., Shafie A.A., Lee V.K.M., Abdul-Rahman T.H. (2014). Study protocol of empower participatory action research (empower-par): A pragmatic cluster randomized controlled trial of multifaceted chronic disease management strategies to improve diabetes and hypertension outcomes in primary care. BMC Fam. Pract..

[80] Bachmann M.O., Bateman E.D., Stelmach R., Cruz Á.A., de Andrade M.P., Zonta R., Zepeda J., Natal S., Cornick R., Wattrus C. (2018). Integrating primary care of chronic respiratory disease, cardiovascular disease, and diabetes in Brazil: Practical Approach to Care Kit (PACK Brazil): Study protocol for randomized controlled trials. J. Thorac. Dis..

[81] Van Meel C., Kidd M.R. (2018). Why strengthening primary health care is essential to achieving universal health coverage. CMAJ.

[82] Palagyi A., Dodd R., Jan S., Nambiar D., Joshi R., Tian M., Abimbola S., Peiris D. (2019). Organisation of primary health care in the Asia-Pacific region: Developing a prioritised research agenda. BMJ. Global. Health.

[83] Hirschhorn L.R., Langlois E.V., Bitton A., Ghaffar A. (2019). What kind of evidence do we need to strengthen primary healthcare in the 21st century?. BMJ. Global. Health.

[84] Löckenhoff C.E., De Fruyt F., Terracciano A., McCrae R.R., De Bolle M., Costa P.T., Aguilar-Vafaie M.E., Ahn C.K., Ahn H.N., Alcalay L. (2009). Perceptions of aging across 26 cultures and their culture-level associates. Psychol. Aging..

[85] Slobodin O., Clempert N., Kula Y., Cohen O. (2020). Educating health professionals for cultural competence in emergency situations: A study protocol for a randomized controlled trial. J. Adv. Nurs..

[86] Ginzburg C., Ankarloo B., Henningsen G. (1990). Deciphering the sabbath. Early Modern European Witchcraft: Centres and Peripheries.

[87] Mack A. (1991). In Time of Plague: The History and Social Consequences of Lethal Epidemic Disease.

[88] Porter R., Bourriau J. (1992). The case of consumption. Understanding Catastrophe.

[89] US Department of Homeland Security Homeland Security Presidential directive 21. Public Health and Medical Preparedness. October 2007. https://fas.org/irp/offdocs/nspd/hspd-21.htm.

[90] Schoch-Spana M., Courtney B., Franco C., Norwood A., Nuzzo J.B. (2008). Community resilience roundtable on the implementation of Homeland Security Presidential Directive 21 (HSPD-21). Biosecur. Bioterror..

